# Editorial: Recent Advances in the Research of Cerebrovascular Aging

**DOI:** 10.3389/fnagi.2020.00180

**Published:** 2020-06-24

**Authors:** Franciska Erdő

**Affiliations:** Faculty of Information Technology and Bionics, Pázmány Péter Catholic University, Budapest, Hungary

**Keywords:** aging, cerebrovascular reactivity, small vessel disease, blood-brain barrier, efflux transporters, fMRI, cognitive assessment, cerebral ischemia

In our special issue the different aspects of cerebrovascular aging have been discussed. In the original article authored by Bálint et al. global forebrain ischemia and later reperfusion were induced by the bilateral occlusion and later release of both common carotid arteries in young and aged Sprague-Dawley rats. Spreading depolarizations were elicited repeatedly with topical 1 M KCl. Pial vascular density was measured in green intrinsic optical signal images of the brain surface, while the density and resting diameter of the cortical penetrating vasculature was estimated with micro-computed tomography of paraformaldehyde-fixed cortical samples. Pial arteriolar dilation was found to be reduced in the old rat brain in response to spreading depolarization or ischemia induction. Spreading depolarization was recognized as a potent pathophysiological contributor to ischemic lesion expansion, in part because of the insufficiency of the associated cerebral blood flow response. The age-related impairment of cerebral vasoreactivity as shown by Bálint et al. is suggested to contribute to the age-related acceleration of ischemic lesion development.

After cerebral ischemia, the ratio between astroglial cells and neurons in the neurovascular unit is disrupted in the perilesional area. Gresita et al. hypothesized that restoring the balance within the neurovascular unit may lead to an improved neurorestoration after focal ischemia. A novel, innovative technology was used for reprogramming of reactive glia into neuroblasts and mature neurons by a retroviral delivery system encoding the transcription factor Ngn2 alone or in combination with the antiapoptotic factor Bcl-2 to target proliferating astrocytes in the neocortex in young and aged mice after cerebral ischemia. The conversion efficacy of proliferating astrocytes into neurons after cerebral ischemia was however, disappointingly low. The authors hypothesized that therapeutic vectors carrying the conversion gene were engulfed by phagocytes shortly after intracortical administration. Gresita et al. suggested to use other viral vectors and combinations of transcription factors to improve the efficacy of glia-to-neuron conversion after stroke in young and aged rodents.

Miller et al. described a clinical study on the measurement of the effect of advanced age on cerebrovascular reactivity. Young and older healthy, physically active adults with low vascular risk participated in the study. Cerebrovascular reactivity was measured in response to hypercapnia using 4D flow MRI, which allows for simultaneous angiographic and quantitative blood flow measurements in the intracranial arteries [right and left internal carotid arteries (ICA), right and left middle cerebral arteries (MCA) and basilar artery (BA)]. As it was shown older adults had lower global cerebrovascular reactivity and reduced reactivity was also shown individually in multiple intracranial arteries compared with young adults. In addition, the MCA dilated significantly in response to hypercapnia in young, but not older adults, which is a marker or predictor of future risk of cerebrovascular disease. These results demonstrated using 4D flow MRI, that normal aging is associated with lower cerebrovascular reactivity in healthy adults and also demonstrated that the state-of-the-art technology of 4D flow MRI may provide a promising new alternative to measure cerebrovascular physiology without the limitations of commonly used techniques.

In another clinical study performed by Zhao et al. three groups of aged patients were compared. The first group has a severe small vessel disease, the second has a low severity small vessel disease and the third was an aged –matched healthy group. A cognitive behavioral test was performed to evaluate the possible cognitive deficit of the patients. The digital clock drawing test (dCDT) has been proved to be a more useful assessment tool for cognitive disorders compared to traditional clock drawing test DT (tCDT). Zhao et al. aimed to check whether this tool worked well in capturing some specific aspects of cognitive performance in aged patients with small-vessel disease. The dCDT and a series of neuropsychological assessments were performed to evaluate the cognitive function of participants. Severe-small vessel diseased patients showed higher air-time percentage and lower mean handwriting/drawing pressure on surface during drawing compared with low-severity and healthy subjects. The data indicated that some early manifestations of cognitive deficits in aged patients with small vessel disease could be detected using the dCDT with a brand-new perspective different from the tCDT.

Resting state fMRI is increasingly used to unravel the functional neuronal networks in health and disease. In particular, this technique of simultaneously probing the whole brain has found high interest in monitoring brain wide effects of cerebral disease and in evaluating therapeutic strategies. Such studies, applied in preclinical experimental mouse models, often require long-term observations. These long periods of following the functional deficits during disease evolution as well as the functional recoveries during therapeutic interventions represent a substantial fraction of the life span of the experimental animals. Based on this observation Egimendia et al. described a resting state fMRI study on different groups of C57Bl6 mice of varying age between 2 and 13 months without any therapeutic or surgical intervention. Dedicated data analysis resulted in an inverse U-shape curve of functional connectivity strength in both the sensorimotor and default mode network. This inverse U-shape pattern presented a maximum of functional connectivity strength at 8–9 months of age, followed by a continuous decrease during later aging phases. At progressed aging at 13 months, the reduction of connectivity strength was ~50%. The authors concluded that these substantial age dependent changes in functional connectivity strength must be considered in future longitudinal studies.

During the last decade several research article reported a relationship between advanced age and changes in the integrity of the blood-brain barrier. These alterations manifested not only in the morphology and structure of the cerebral microvessels but also in the expression and functionality of the transporter proteins in the apical and basolateral surface of the capillary endothelial cells. Age-associated downregulation of the efflux pumps (ABC transporters) resulted in increased permeability and greater brain exposure to different xenobiotics enhancing the risk of drug-induced toxicity. In age-related neurodegenerative pathologies like Alzheimer's disease the amyloid beta clearance decreased due to P-glycoprotein dysfunction leading to higher brain exposure. In contrast, in stroke an enhanced P-glycoprotein function was reported in the cerebral capillaries making even more difficult to perform effective neuroprotective therapy in the infarcted brain area. The mini-review of Erdo and Krajcsi is focusing on the role of efflux transporters at the blood-brain barrier in age-related brain pathologies and also in healthy aging.

In summary, this special issue presents a wide spectrum of original research studies from animal experiments to clinical investigations focusing on different aspects of cerebrovascular aging. Some innovative methodological and evaluation-related approaches are nicely discussed.

## Author Contributions

The author confirms being the sole contributor of this work and has approved it for publication.

## Conflict of Interest

The author declares that the research was conducted in the absence of any commercial or financial relationships that could be construed as a potential conflict of interest.

